# Effects of Psychological Empowerment–Based Motivational Interviewing Program on Self-Management Behavior in Patients With Early Chronic Kidney Disease: A Mixed Methods Study

**DOI:** 10.1155/jonm/6822744

**Published:** 2025-11-21

**Authors:** Yi Cui, Jiayao Li, Na Liu, Rong Li, Xiaoyan Li, Fuqiong Li, Shuang Zhai, Yinling Zhang, Hongbao Liu, Honghong Lv

**Affiliations:** ^1^Department of Nephrology, Tangdu Hospital, The Fourth Military Medical University, Xi'an, China; ^2^Department of Nursing, The Fourth Military Medical University, Xi'an, China; ^3^Department of Nephrology, Xijing Hospital, The Fourth Military Medical University, Xi'an, China

**Keywords:** chronic kidney disease, ego depletion, mixed methods, motivational interviewing, psychological empowerment, randomized controlled trial, self-management behavior

## Abstract

**Background:**

Chronic kidney disease (CKD) is a global health threat to patients' physical and mental health. Effective self-management can slow disease progression in early stages. However, prolonged treatment often leads to ego depletion and subsequently impacts self-management. Interventions to address this issue remain underdeveloped.

**Aim:**

To evaluate the effects of psychological empowerment–based motivational interviewing program on early-stage CKD patients' self-management, perceived empowerment, and ego depletion and to explore their engagement experiences and the underlying reasons for the intervention's effectiveness.

**Methods:**

The study employed the explanatory sequential mixed methods design comprised of a randomized controlled trial and a qualitative study, which were conducted in a tertiary hospital from July 2022 to November 2023. About 70 patients with early CKD were randomly assigned to a control group (*n* = 35) receiving standard clinical nursing, or an intervention group receiving a 12-week nurse-led psychological empowerment–based motivational interviewing program consisting of four interview sessions and four consolidation sessions. CKD Self-Management Behavior Scale, Patient Perception of Empowerment Scale, Self-Regulation Fatigue Scale, and biochemical indicators were collected at baseline (T1), after 4 weeks of intervention (T2), immediately postintervention completion (T3), and 4 weeks after intervention completion (T4). Data were analyzed by generalized estimating equation model. Semistructured interviews were conducted with the participants in the intervention group.

**Results:**

The participants' mean age was 42.76 years (SD = 10.59). Compared with the control group, the intervention group had a statistically significant improvement in self-management behavior (T2: *β* = 18.26, T3: *β* = 23.73, T4: *β* = 23.78; *p* < 0.001), ego depletion (T2: *β* = −8.46, T3: *β* = −11.35, T4: *β* = −13.35; *p* < 0.001), and perceived empowerment (T2: *β* = 5.77, *p*=0.002; T3: *β* = 9.41, T4: *β* = 8.99; *p* < 0.001). Qualitative interviews of 14 participants indicated that the intervention could affect self-perception, improve emotion, and establish healthy behaviors, which may explain such encouraging effects.

**Conclusions:**

The psychological empowerment–based motivational interviewing program produced immediate and delayed benefits on self-management, perceived empowerment, and ego depletion in patients with early CKD. These findings provide new strategies for chronic disease management and psychological nursing.

**Trial Registration:** Chinese Registry of Clinical Trials: ChiCTR2200064257.

## 1. Introduction

Chronic kidney disease (CKD) is a global public health issue with a high prevalence of approximately 13.4% worldwide and 10.8% in China [1, 2]. As kidney function deteriorates, the risk of various complications, cardiovascular events, and mortality significantly increases among CKD patients [3]. This presents formidable challenges to personal health and societal progress, underscoring the urgent need for effective control and management of CKD.

The management of CKD necessitates lifelong self-care for the majority of patients, thereby emphasizing the significance of self-management. Currently, studies have shown that effective self-management in early CKD patients can significantly reduce complications and slow disease progression [4]. For instance, patients with good self-management often adhere to a kidney-specific diet, which helps control blood pressure and reduce proteinuria, thereby slowing the decline in glomerular filtration rate [5]; consistently taking prescribed medications is also crucial for managing hypertension and proteinuria, which are key factors in the progression [6]; regularly monitoring blood pressure and identifying signs of worsening conditions such as edema and fatigue enable early detection of complications and timely treatment [7]. However, factors such as recurrent illness and high costs often lead to negative emotions during the course of CKD treatment [8]. The expression of these negative emotions depletes patients' psychological resources or energy, resulting in ego depletion. This state of diminished self-control resources impairs an individual's ability to regulate cognition, decision-making, and behavior, ultimately affecting their self-management behavior [9]. Furthermore, the asymptomatic nature of early-stage CKD presents a fundamental challenge. Patients often fail to recognize the severity of their condition and thus exhibit suboptimal self-management behaviors [10]. This stems from the lack of physical symptoms, which impairs the formation of a strong disease representation or perceived threat. As outlined in the common-sense model of self-regulation, patients are more likely to activate disease-related information and take action against health threats when physical symptoms are noticeable. In the absence of symptomatic cues such as pain or discomfort, patients may downplay the severity of the disease, resulting in low perceived susceptibility. They might perceive CKD as lacking serious complications and immediate urgency, thereby reducing motivation. The perceived future benefits could be outweighed by the immediate costs and burdens of self-management [11]. Although some interventions such as health education utilizing diverse modern communication software, continuous nursing, and cloud platform health management are valuable, they predominantly concentrate on improving patients' behavior from an external behavioral guidance through didactic education and reminder systems [12, 13]. Therefore, the deep exploration of patients' psychological potential, the fundamental stimulation of their motivation to take responsibility for their health, and the subsequent improvement in self-management behavior have become crucial issues in providing clinical psychological nursing for early CKD patients.

Psychological empowerment theory refers to a psychological state or cognitive complex in which individuals experience empowerment by acquiring positive and valuable experiences from activities that can generate motivation and satisfaction [14]. It posits that fostering four key dimensions: meaning (personal relevance of behavior), competence (self-efficacy), self-determination (autonomy), and impact (belief that actions matter), which can enable individuals to transition from a passive to an active role in their health, thereby motivating sustained self-management [15]. The World Health Organization (WHO) has emphasized the integration of psychological empowerment theory into nursing care for patients with chronic diseases, enabling them to self-manage their condition and actively contribute toward improving disease outcomes and quality of life [16]. The concept of psychological empowerment provides theoretical guidance, but its practical application requires integration with specific behavioral intervention methods to achieve optimal outcomes [17].

Motivational interviewing is a specialized technique that primarily facilitates individuals in identifying existing or potential issues through interviews, while delving deeply into and effectively utilizing patients' intrinsic motivation during the process of behavioral enhancement [18]. Research conducted on patients with alcohol addiction, physical activity, and other conditions has revealed that motivational interviewing can enhance individuals' psychological well-being and improve self-care abilities [19, 20]. Motivational interviewing employs open-ended questions and reflective listening to help patients explore the significance behind behavior change, identify their reasons for change, and develop a sense of self-determination. It also builds self-efficacy by affirming strengths and recalling past successes. Furthermore, it enhances autonomy and self-worth by exploring ambivalence and supporting self-defined goals [18]. However, previous studies on motivational interviewing have focused primarily on external behavioral change, with limited exploration of deep psychological factors, and its effectiveness in patients with early CKD remains uncertain. On this basis, the present study implements interventions based on psychological empowerment theory, in conjunction with motivational interviewing. Psychological empowerment theory is derived from the motivational pathway, while motivational interviewing offers practical techniques for fostering motivation. This combination represents a novel approach to addressing the motivational deficits in early CKD patients and to enhancing self-management behavior.

Therefore, this study aimed to validate the effect of psychological empowerment–based motivational interviewing program in enhancing self-management behavior among patients with early CKD. We hypothesized that, compared with the control group, patients in the intervention group would have (1) enhanced self-management behavior (primary outcome), (2) improved perceived empowerment, (3) reduced ego depletion, and (4) improvements in blood urea nitrogen (BUN), serum creatinine (Scr), blood uric acid (BUA), and estimated glomerular filtration rate (eGFR) after 12 weeks. Furthermore, this study explored patients' real engagement experiences and perceived effects during the intervention process while analyzing the underlying reasons for their improvement in self-management behavior.

## 2. Materials and Methods

### 2.1. Study Design

This mixed methods study comprised a prospective single-blinded, parallel-group, randomized controlled trial (RCT), and a descriptive qualitative study. Specifically, the study employs an explanatory sequential design, prioritizing a quantitative approach to provide robust evidence on the effectiveness of the multicomponent intervention program. Subsequently, the qualitative analysis is essential to explain how the intervention produced its effect, understand participants' experiences in depth, and identify the mechanisms of change from the participants' perspectives. Such a design is especially well-suited for the present study because this integration of data provides a more comprehensive understanding of the intervention's impact than either approach could offer alone [21].

The report conformed to the Consolidated Standards of Reporting Trials (CONSORT) guidelines [22]. This study has been approved by the Ethics Committee of Air Force Medical University (No. 202206-02). The confidentiality of the study process, nondisclosure of patient privacy, and utilization of data solely for scientific research purposes are principles we firmly adhere to.

### 2.2. Participants

Participants were recruited from patients with early-stage CKD who were hospitalized in the Department of Nephrology, the Affiliated Hospital of Air Force Medical University from July 2022 to November 2023. Potential participants were identified by consecutively screening the electronic medical records of all patients during the study period. Those who met the eligibility criteria were approached by a research nurse, who explained the study details. The inclusion criteria were as follows: (1) meeting the diagnostic criteria for CKD as defined by the Kidney Disease: Improving Global Outcomes (KDIGO) 2012 clinical practice guideline [23]; (2) having primary glomerular disease with a duration of ≥ 3 months; (3) being at clinical Stages 1–3, as determined by eGFR calculated using the CKD-EPI equation and persistent albuminuria (UACR ≥ 30 mg/g) where applicable; (4) being aged ≥ 18 years old; (5) possessing normal communication skills and proficiency in using mobile phones and WeChat applications; and (6) providing informed consent and voluntarily participating in the study. The exclusion criteria included the following: (1) having cognitive impairment or comorbid mental illness; (2) being complicated with cardiovascular, nervous system, pulmonary, and other diseases; (3) participation in other concurrent clinical trials; (4) experiencing deterioration of condition during the intervention period requiring changes to treatment plans such as dialysis or kidney transplantation; and (5) voluntary withdrawal or loss to follow-up.

According to the formula for comparing the means of two samples: *n*_1_=*n*_2_=2[(*μ*_*α*_+*μ*_*β*_)/*δ*/*σ*]^2^+1/4*μ*_*α*_^2^; typically, *α* is set at 0.05, and *β* is set at 0.10. In this study, the estimated self-management behavior score from the pilot experiment yielded a sample size of *n*_1_=*n*_2_ = 27 participants initially obtained. Considering a potential loss rate of 20%, it was ultimately determined that *n*_1_=*n*_2_ = 35 participants were required to achieve a final total sample size of 70 participants.

For the qualitative phase, objective sampling was employed to select participants in the intervention group based on the principle of maximum differentiation, including factors such as gender, age, education level, disease duration, and CKD stage. The sample size for qualitative research adhered to the standard of data saturation, the point at which no new themes or insights are generated from additional data [24]. Saturation was considered achieved when three consecutive interviews yielded no new codes or themes relevant to the research objectives [25]. Ultimately, a total of 14 participants were interviewed and identified as A-N according to the interview sequence.

### 2.3. Randomization

The randomization process was conducted by a nonresearch team member using a computerized random-number generator at a 1:1 assignment ratio. Allocation details were then sealed in identical opaque envelopes by a nurse who had no direct involvement in the trial. Baseline assessments were performed by a researcher (with no direct relationship with the participant) upon admission. One researcher subsequently opened a sealed envelope and assigned the participant to their respective group. During the qualitative phase, participants from the intervention group were purposefully selected for individual semistructured interviews.

### 2.4. Blinding

The randomization assignment was kept undisclosed to all individuals prior to the commencement of the intervention. Given the nature of the intervention, blinding was not feasible for the participants in the intervention group and researchers providing the intervention, but outcome assessors and statistical analysts were blinded to control for subjective bias. All study team members and participants and their families were asked to avoid discussing the trial process and results with each other.

### 2.5. Study Intervention

#### 2.5.1. Intervention Group: Psychological Empowerment–Based Motivational Interviewing Program

Firstly, the research team members underwent intensive study and training in theoretical knowledge and techniques related to this study through theoretical teaching and practical application of intervention technology. Motivational interviewing is a patient-centered specialized interview technique that facilitates patients in exploring and resolving psychological conflicts, harnessing their inherent potential and resources, and fostering internal psychological motivation. The intervention program integrates the principles of psychological empowerment theory with motivational interviewing, employing effective communication skills such as empathetic expression and open-ended questioning to bolster patients' self-efficacy, ignite their intrinsic motivation toward assuming responsibility for their own health, and supplementing and sustaining their psychological reservoirs and vitality. The preliminary psychological empowerment–based motivational interviewing program for patients with early CKD was formulated through a synthesis of literature review from multiple sources, such as the authoritative motivational interviewing website (http://zh-cn.motivationalinterviewing.org) [26], video, and books including ‘Building Motivational Interviewing Skills: A Practitioner Workbook' [27], ‘Motivational Interviewing: Helping people change' [18], and ‘Motivational Interviewing in Health Care: Helping Patients Change Behavior' [28]. In addition, we synthesized research on chronic disease management incorporating principles of psychological empowerment and motivational interviewing [16, 19, 20], and adapted the core components of the clinical context of early-stage CKD patients through research team discussion until a consensus was reached.

Then, the preliminary program was revised to develop the final psychological empowerment–based motivational interviewing program through two rounds of Delphi method (Supporting Information 1). As detailed in Table 1, the intervention program spanned a duration of 12 weeks and consisted of two stages. The first stage encompassed the interview phase, which was conducted once weekly for a total of four sessions. Each week focused on specific topics: guiding: establishing the foundation of relationships; focusing: planning for direction; calling out: preparing for change; and planning: bridging to change. Face-to-face interviews were conducted during hospitalization, while one-to-one telephone interviews took place after discharge. Each interview session was limited to a controlled timeframe ranging from 20 to 30 min. The second stage involved effect consolidation and occurred once every 2 weeks over four sessions. The follow-up guided by motivational interviewing was conducted primarily via telephone or WeChat, with each session being limited to a duration of 10–20 min. The nurse-led intervention program was administered by the primary researcher (the first author), an experienced registered nurse with extensive experience in CKD management, formal training in motivational interviewing, and proficiency in the principles of psychological empowerment. To ensure intervention fidelity and quality, the implementation process was supervised and guided by a multidisciplinary team including a nursing professor specializing in psychological counseling, the director, and head nurse of the Nephrology Department. The intervention process adhered to the TIDieR checklist (Supporting information 2).

#### 2.5.2. Control Group

Patients received standard clinical nursing, including the collection of general information and psychological status upon admission, establishment of treatment and nursing files, disease monitoring, and health education. After discharge, the researchers followed up the patients via telephone every 2 weeks, 10–20 min each time, lasting for 12 weeks. The follow-up content was structured, mainly including their recent physical conditions and potential negative emotions. Patients in the control group and intervention group were arranged in different wards to minimize contamination as much as possible. Researchers and patients signed confidentiality agreements to prevent communication about trial content.

### 2.6. Outcome Measures

The primary outcome was the self-management behavior of CKD patients, while the secondary outcomes included perceived empowerment, ego depletion, and disease-related biochemical indicators (BUN, Scr, BUA, and eGFR). All outcomes were assessed at baseline (T1), after 4 weeks of intervention (T2), immediately postintervention completion (T3), and 4 weeks after intervention completion (T4). The specific measurement tools and their key properties are summarized in Table 2 [29–34]. Sociodemographic data such as age, gender, marital status, and payment method for medical expenses, along with disease information including CKD stage, duration of disease and review frequency were collected at T1 only.

Two research assistants, who served as the primary instruments in the collection of qualitative data, were trained by the first author. Prior to the interviews, the researchers were fully trained in qualitative methodologies and possessed experience in CKD nursing. To ensure consistency across interviews, a semistructured interview guide was developed based on the research objectives and a literature review. Then, the draft was revised following discussion with an expert panel comprising two experienced nursing professors, a clinical nursing specialist, and a nephrology nurse. Pilot interviews with two participants were conducted to assess the comprehensibility and feasibility of the guide. The initial draft was further refined based on participant feedback, analysis of the interview data, and evaluation of the interview process, resulting in the final version of the interview guide (Table S1 of Supporting Information 3).

### 2.7. Data Collection Procedures

The data collection through questionnaires was conducted by a researcher who remained unaware of the group assignments. Questionnaires are typically completed during patients' hospital visits for reexamination or via Wenjuanxing (https://www.wjx.cn/). Disease-related biochemical indicators such as BUN, Scr, BUA, and eGFR were analyzed by reviewing computer records from the hospital. To minimize bias, no monetary incentives were offered to participants because financial rewards may distort data accuracy by introducing selection or reporting bias [35]. Survey responses were also strictly anonymous, and no personally identifiable information was collected to mitigate social desirability bias [36].

The qualitative study commenced 4 weeks after intervention completion. The researcher and interviewees mutually agreed upon a suitable time and location. Each interview session lasted between 20 and 30 min. Face-to-face semistructured interviews were conducted in an unoccupied room within the nephrology inpatient department during nontreatment hours. During the interview, the patients were informed in advance that they needed to record the whole process, and after obtaining consent, the whole process was recorded with a voice recorder. They were encouraged to openly express their thoughts while being attentively listened to without interruption. Additionally, nonverbal cues such as vocal intonation, facial expressions, body movements, and emotional changes were diligently observed and recorded throughout the conversation. At the end of each interview session, patients were asked if they had any additional comments or if a subsequent interview might be necessary.

### 2.8. Statistical Analysis

The quantitative study data were analyzed using IBM® SPSS Statistics Version 27, and all analyses adhered to the intention-to-treat principle [37]. Categorical variables are presented as frequencies and percentages, and continuous variables are presented as means with standard deviations (SDs). The statistical methods employed included the chi-square test, one-way analysis of variance, and independent sample *t*-test. A generalized estimating equation model was utilized to determine between-group differences in changes in self-management behavior, perceived empowerment, ego depletion, BUN, Scr, BUA, and eGFR from T1 to T4. This model accounts for internally correlated repeated measures and allows for missing data due to incomplete or dropped out follow-up without requiring imputation of missing data in longitudinal studies; it is particularly suitable for intention-to-treat analyses [38]. To control for possible confounding factors, we set age, gender, marital status, education level, and monthly household income as covariates [39]. A standardized mean difference (Cohen *d*) was calculated post hoc using the model's regression coefficient for the intervention effect divided by the baseline SD of the outcome measures, with a value less than 0.2 indicating a negligible effect, 0.2 ≤ *d* < 0.5 indicating a small effect, 0.5 ≤ *d* < 0.8 indicating a moderate effect, and *d* ≥ 0 .8 indicating a large effect [40]. The level of statistical significance was set at a 2-sided *p* value less than 0.05.

For qualitative data, the first author and research assistant transcribed the recorded data within 24 h following the conclusion of each interview. They engaged in repeated listening and cross-checking to ensure the integrity, authenticity, and accuracy of the transcriptions. The data were analyzed using the classical phenomenological Colaizzi analysis method [41]: reading and rereading patients' expressed content to develop a comprehensive understanding, extracting pertinent statements aligned with the research question, refining meaningful statements, organizing extracted statements into thematic groups, linking these themes back to individuals for detailed descriptions, synthesizing similar perspectives and proposing thematic concepts, and returning the results to patients for verification.

## 3. Results

**Figure 1:**
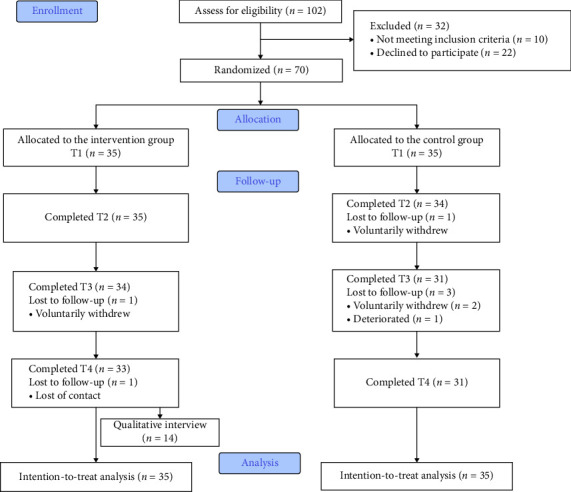
CONSORT flow diagram.

A total of 102 patients with early CKD were assessed for eligibility, and 70 patients were randomly allocated into two groups, with 35 patients in each group. Finally, 33 patients completed the study in the intervention group (1 patient voluntarily withdrew and 1 patient was lost to follow-up), while the control group included 31 patients (1 patient deteriorated and 3 patients voluntarily withdrew). Overall, a total of 64 patients completed the study, resulting in a participation rate of 91.43%.  Figure 1 illustrates the CONSORT flow diagram.

### 3.1. Participants Characteristics

The mean age of the participants was 42.76 years (SD = 10.59), and 57.14% were male. The intervention group consisted of 19 males and 16 females, with an average age of 40.91 (9.95) years. In comparison, the control group comprised 21 males and 14 females, with an average age of 44.83 (9.66) years. Before the intervention, the results indicated no significant differences in sociodemographic characteristics, disease information, or outcome variables at baseline between the two groups (*p* > 0.05). Thus, the sociodemographic characteristics, disease information, and outcome variables for both groups were balanced and comparable, as presented in Table 3.

### 3.2. Effects on the Primary Outcomes

**Figure 2:**
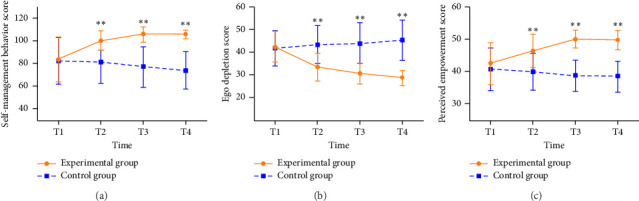
Mean changes in scores of self-management behavior (a), ego depletion (b), and perceived empowerment (c) over time (*n* = 70).

Compared with the control group, the intervention group had a statistically significant improvement in the total score of self-management behavior from T1 to the endpoints at T2, T3, and T4 (T2: *β* = 18.26; 95% CI [6.41, 30.10], *p* < 0.001; T3: *β* = 23.73; 95% CI [12.26, 35.20], *p* < 0.001; T4: *β* = 23.78; 95% CI [12.69, 34.87], *p* < 0.001), with the effect size (Cohen *d*) = 0.90 to 1.52. The changes in self-management behavior are presented in Table 4 and illustrated in Figure 2(a).

### 3.3. Effects on the Secondary Outcomes

Compared with the control group, the intervention group had a statistically significant improvement in the total score of ego depletion from T1 to the endpoints at T2, T3, and T4 (T2: *β* = −8.46; 95% CI [-13.67, −3.25], *p* < 0.001; T3: *β* = −11.35; 95% CI [-16.13, −5.56], *p* < 0.001; T4: *β* = −13.35; 95% CI [−17.88, −8.82], *p* < 0.001), with the effect size (Cohen *d*) = 1.46 to 2.37.

Compared with the control group, the intervention group had a statistically significant improvement in the total score of perceived empowerment from T1 to the endpoints at T2, T3, and T4 (T2: *β* = 5.77; 95% CI [1.39, 10.15], *p*=0.001; T3: *β* = 9.41; 95% CI [5.65, 13.16], *p* < 0.001; T4: *β* = 8.99; 95% CI [5.21, 12.77], *p* < 0.001), with the effect size (Cohen *d*) = 0.74 to 1.47. The changes in ego depletion and perceived empowerment are presented in Table 4 and illustrated in Figures 2(b) and 2(c).

However, for Scr, BUN, BUA, and eGFR, there is no statistically significant difference between the two groups from T1 to the endpoints at T2, T3, and T4. The comparison of the variables is presented in Table 4.

### 3.4. Engagement Experience and the Underlying Reasons for the Effect of Psychological Empowerment–Based Motivational Interviewing Program

A total of 14 participants completed the qualitative interview (when the interview reached the 12th participant, the data reached saturation, and 2 participants were still interviewed). The participants were labeled as A ∼ N based on their interview order, with an average age of 41.00 years and an average interview time of 27.5 min. Three themes emerged from the interview data: (i) changes in self-perception, (ii) improvement of emotions, and (iii) establishment of health behavior. The participants' characteristics are shown in Table S2 of Supporting Information 3. The three themes, 12 subthemes, and supporting quotes are shown in Table S3 of Supporting Information 3. This report adheres to the COREQ statement outlined in Table S4 of Supporting Information 3.

#### 3.4.1. Theme 1: Changes in Self-Perception

##### 3.4.1.1. Enhancement of Self-Efficacy

After undergoing the intervention, patients believed that they had the ability of self-management and exhibited heightened confidence in effectively controlling the progression of CKD. For example, Participant A: “I think the communication … has really increased my confidence … I can obviously change some problems …This process of learning is gradual but invaluable. Now I have learned a lot about the management of chronic diseases, and I still believe I can apply them in my daily life.”

##### 3.4.1.2. Reinforcement of Self-Acceptance

The patients reported that their participation facilitated a gradual acceptance of the current reality and enabled them to approach the disease with a positive mindset. For example, Participant E: “I have now fully accepted the fact … it is no use complaining all the time. The most important thing is that I have to make some changes and make some behaviors …. It's better to be cooperative than to say anything. The more positive you are to face it, the more you find that you seem to have inexhaustible energy.”

##### 3.4.1.3. Improvement of Self-Identity

The patients perceived that the intervention engendered a sense of being valued and acknowledged for their actions, thereby fostering heightened enthusiasm toward life and cultivating more defined aspirations for the future. For example, Participant L: “Love others must first love yourself … I must not give up. I must have the courage to live with the disease … my life is still full of value. I also keep pushing myself to learn, exercise, and grow, and I always take responsibility for my own health.”

##### 3.4.1.4. Accurate Comprehension of Disease

The intervention is beneficial in rectifying patients' misconceptions about the disease, facilitating accurate comprehension of CKD, and alleviating their fear toward it. For example, Participant I: “This disease is difficult to completely cure, then I will face it head-on, listen to the doctors and nurses, treat it well, and control the disease. My only idea now is to stick to the treatment … to minimize the damage of the kidney disease and control the progress.”

##### 3.4.1.5. Enhanced Recognition of Individual Capabilities

The intervention enables patients to recognize and harness their personal, familial, social, and other available resources. This approach fosters an environment that empowers patients to regain confidence in confronting their illness and enhances their ability for self-management. For example, Participant F: “A large part of the power comes from friends around me. Now the hospital … not only treating our physical diseases but also providing us with popular science and health education. Nurses … is also a great support to us. Honestly, if you hadn't communicated with me all this time, I don't think my mindset would have changed so quickly … now I believe that everything will be better. I should encourage myself more and want to do more positive and happy things.”

##### 3.4.1.6. Comprehension of the Meaning of Life

The intervention facilitates patients' exploration of life's significance, enhances their determination to persevere, and instills renewed hope for their existence. For example, Participant D: “I am genuinely delighted that my parents change their ways to cook me all kinds of delicious food every day, my husband is very supportive of me in all aspects … my parents-in-law will not look down on me … if I don't take care of my body, it is really a bit impossible, even if it is not for myself, I want to live for them too … I have plans to have a baby, and I need to get my body to try not to interfere with my birth later.”

#### 3.4.2. Theme 2: Improvement of Emotions

##### 3.4.2.1. Reduction in Negative Emotions

Through the intervention, patients reported a reduction in their usual fear, anxiety, impatience, and other negative emotions associated with the disease, leading to an enhanced sense of cheerfulness and relaxation in their lives. For example, Participant C: “I used to be very anxious … (sighed). Through this interview, I found that I thought the disease was too serious … it seemed that I was not different from others. I feel that while I am still in the early stage, I can control it, and a positive attitude is still important.”

##### 3.4.2.2. Elicitation of Positive Emotions

The intervention elicits heightened positive emotions in patients, fosters an optimistic approach toward their illness, and enhances patient motivation to engage in self-management behaviors. For example, Participant D: “I will not give myself too much pressure now. I am happy every day and feel that all the problems are not problems.” Participant J: “I think the main mentality of my own is quite good … but also very optimistic, not to get stuck in rut, I now full of confidence.”

##### 3.4.2.3. Courage to Articulate Emotions

The intervention facilitates patients' courageous expression of their feelings and emotions, alleviates negative emotions, and mitigates their psychological burden. For example, Participant M: “I will go to the park to take a walk, relax the mood, release the pressure, I feel this method is quite suitable for me, sometimes I can regulate my own mood, and do not have to be afraid of affecting the family and friends around me.”

#### 3.4.3. Theme 3: Establishment of Health Behavior

##### 3.4.3.1. Active Learning Disease-Related Knowledge

The intervention facilitates patients' recognition of the significance of acquiring disease-related knowledge, thereby enhancing their motivation to engage in learning. For example, Participant H: “I have learned a lot about CKD. I also began to learn about kidney disease diet. I took the initiative to follow some public accounts, buy books, and read popular science by some experts, to get along better with kidney disease and reduce the burden on my family.”

##### 3.4.3.2. Modification of Lifestyle

After participating in the intervention, patients began to improve their lifestyle habits, including diet, work-life balance, physical activity, and assume accountability for their own health. For example, Participant B: “I now go to work normally every day, and dare not stay up late at night. My work and rest are much more regular than before … sometimes I can't bear to eat more, but I have consciously controlled it. I used to smoke, and now I know I can't.”

##### 3.4.3.3. Development of Interests and Hobbies

Through the intervention, patients acquired the ability to cultivate their interests and hobbies, thereby enhancing their quality of life and fostering a sense of enthusiasm and passion for living. For example, Participant K: “I will also take more time to appreciate the small joys of life and handle other things at a leisurely pace. I go to the flower and bird market to buy some flowers or potted plants … I also plan to take a class to learn how to arrange flowers. I really enjoy flowers and plants.”

## 4. Discussion

This study combined psychological empowerment theory and motivational interviewing for the first time. Psychological empowerment theory serves as a foundational framework for understanding the development of motivation, whereas motivational interviewing offers practical techniques for fostering motivation. The psychological empowerment–based motivational interviewing program was initially implemented in patients with early CKD. The findings demonstrated that this intervention effectively enhanced patients' self-management behavior and perceived empowerment while also mitigating their ego depletion. The qualitative findings were consistent with the quantitative findings, as patients experienced a shift in self-perception, positive improvement in mood, and actively established behaviors responsible for their own health, which may provide a more comprehensive explanation of the intervention's effects.

The psychological empowerment–based motivational interviewing program can effectively enhance self-management behavior in patients with early CKD. Over time, there was a gradual increase in the score of self-management behavior, indicating both immediate and delayed effects. Some studies believe that patients' own motivation and beliefs play a crucial role in transforming health education knowledge into behavior change [42]. Motivational interviewing serves as an easily implementable and effective interview technique to achieve targeted behavior change, yielding positive outcomes for patients' self-management behavior [43]. The reasons behind this success lie in several aspects: Firstly, through weekly interviews covering various topics, this intervention positively influences patients' beliefs regarding disease management while promoting their understanding of CKD and improving compliance. Secondly, by employing core techniques such as open-ended questions, development conflicts, and information exchange [44], it becomes possible to comprehend the dilemmas faced by patients, which helps them formulate plans and take necessary actions. Techniques such as affirmation and guiding self-efficacy strengthen active commitment from patients to support behavioral changes [45]. Moreover, patients gain insight into the benefits of enhancing self-management behaviors which stimulate their sense of initiative while respecting their autonomy, ultimately intrinsically boosting motivation for long-term engagement in disease self-management resulting in more evident and lasting effects. Qualitative research findings also reveal that gaining a new perspective on oneself along with perceptions about the disease lays a cognitive foundation for improved self-management behaviors; furthermore, establishing health-related behaviors serves as specific external manifestations of such practices.

The psychological empowerment–based motivational interviewing program can effectively improve the perceived empowerment of patients with early CKD. The score of perceived empowerment gradually increases over time, demonstrating both immediate and delayed effects. By integrating the theory of empowerment into behavioral interventions, it becomes possible to activate individuals' internal potential, improve patients' ability to understand and actively engage in disease management, and foster a sense of autonomous rights awareness [46]. This may be attributed to the fact that during CKD treatment, many patients tend to passively accept information provided by medical staff without independent thinking or self-awareness. They often believe that doctors and nurses are solely responsible for treating their condition. Motivational interviewing based on psychological empowerment incorporates the concept of empowering patients during interviews by mobilizing their knowledge and abilities while granting them decision-making authority [47]. This approach helps boost patients' confidence levels and enhances their capacity for personal development and satisfaction with themselves, ultimately enabling them to take full responsibility for their own health. Furthermore, throughout the interactive process of these interviews, researchers consistently encourage critical thinking among patients while guiding them toward leveraging their individual rights and advantages in managing disease. As a result, this intervention strengthens self-efficacy beliefs and fosters proactive awareness.

The psychological empowerment–based motivational interviewing program can effectively mitigate ego depletion in patients with early CKD. The ego-depletion score exhibits a gradual decline over time, indicating both immediate and delayed effects. Werner and Berkman [48] believe that stimulating the motivation of patients can give full play to their own resources and carry out self-control more effectively to alleviate the loss. This may be attributed to several factors: Motivational interviewing based on psychological empowerment prioritizes patients as the focal point, treating them with equality, respecting their choices and rights, harnessing their subjective initiative, motivating early CKD patients to take responsibility for their own health, effectively mobilizing their psychological resources, and consistently reducing ego depletion [49]. Furthermore, researchers address patient ambivalence through targeted interview questions to help overcome psychological resistance and prevent unnecessary ego depletion. Miracle exploration is also employed to transform individuals or elements important to patients into sources of motivation that strengthen their drive; guiding them toward discovering problem-solving abilities and personal resources while stimulating self-potential serves as compensation for individual-induced ego depletion [50]. Qualitative research findings further validate that through the intervention to excavate the positive feelings of patients and help patients build positive psychological resource, improvements of mood may also be the reason for the reduction of patients' ego depletion. These results suggest that although complete avoidance of ego depletion effects during disease treatment is not feasible, empowering patients and enhancing motivation levels can effectively stimulate patient psychological resources and energy while reducing the impact of ego depletion.

However, there were no significant changes observed in BUN, Scr, BUA, or eGFR before and after the intervention. The reasons for finding may be as follows: Firstly, it is possible that psychological interventions require a longer duration to impact biochemical indicators [51]; secondly, CKD is a lifelong condition where renal function damage cannot be reversed but only controlled [52]. The treatment goals of CKD include delaying further progression of the disease and minimizing renal function impairment. The psychological empowerment–based motivational interviewing program may assist patients in regulating their emotions, reducing ego depletion, improving self-management behaviors, and positively influencing disease stability. Further exploration is needed to understand the underlying reasons behind these findings; therefore, our research team will continue monitoring changes in biochemical indicators within both groups.

## 5. Limitations

Firstly, due to time and manpower constraints, this study only conducted a 12-week intervention and a 4-week follow-up for patients with early CKD, and the biochemical indicators of patients did not change significantly. Future studies should consider extending the intervention period and follow-up duration appropriately, continuously tracking the long-term effects of interventions, and identifying the most suitable timing for interventions. Secondly, the intervention program encompasses multiple components that may require clinical nurses' sufficient time to learn and adapt. And for patients, the inclusion criterion for normal communication skills and proficiency in using WeChat may have excluded older adults or those from disadvantaged backgrounds with limited digital literacy. This limits the generalizability of our findings to the broader CKD population and may introduce selection bias. Future research should explore alternative strategies, such as interactive and simulated interviews powered by artificial intelligence, to enhance the applicability of this intervention program in busy clinical settings and extend its reach to digitally marginalized populations.

## 6. Implications for Nursing Management

Through this study, it is evident that psychological empowerment–based motivational interviewing program exerts positive influences and offers application advantages to chronic disease nursing management. First, both motivational interviewing and psychological empowerment yield positive outcomes by assisting patients in resolving conflicts between internal goals and actual behaviors, instilling hope for the future, and stimulating their potential. Second, psychological empowerment provides theoretical guidance that helps patients strengthen their subjective motivation, deepen their goal expectations, and effectively promote behavior change from a profound psychological perspective. Third, compared to didactic and educational self-management programs for chronic diseases, the person-centered motivational interviewing provided by nurses in the daily environment may achieve more effective and lasting results. Fourth, there were clear principles and techniques to guide the implementation of motivational interviewing, and abundant training resources to help nurses utilize this interview method proficiently. Fifth, nurses can integrate motivational interviewing into routine conversations with patients to establish an equitable nurse–patient partnership, better assist patients in disease treatment participation while empowering them, and internalize the significance of goal achievement and the necessity for behavioral change.

## 7. Conclusions

The present study verified that psychological empowerment–based motivational interviewing program can effectively improve patients' self-management behavior and perceived empowerment while reducing ego depletion. Additionally, it explores the authentic experiences and positive transformations of patients undergoing intervention. Notably, it was found that patients may have self-cognitive changes, mood improvement, and health behavior establishment after intervention. These findings offer a novel perspective for enhancing the quality of clinical psychological nursing for patients with early CKD.

## Figures and Tables

**Table 1:** The specific content of the psychological empowerment–based motivational interviewing program.

Topics	Contents	Notes
Week 1: The first face-to-face motivational interviewing		
Guiding: Establishing the foundation of relationships	• Could you tell me about your current health status? (Please note that patients in the process of expressing their symptoms, actions, or modal particles pay attention to their emphasis and observe patients' understanding of their own disease) ------ Open questions and reflective listening • How would you like your physical transformation to occur within the upcoming 3 months • In the next 3 months, what specific outcomes do you desire for your mental well-being? • What circumstances in life bring you happiness during ordinary times? • Please rate the significance of self-management on a scale from 1 (*not important*) to 10 (*very important*), and provide an explanation for your chosen score. • Kindly rate your level of confidence in self-management on a scale from 1 (*no faith at all*) to 10 (*extremely confident*), and explain the reasoning behind your selected score.	Four elements: Goal, importance, positive feeling, and expectation Intervention dose: 20 to 30 minutes Location: A private, quiet, and dedicated room in Nephrology Department to ensure an environment free from interruptions and distractions

Week 2: The second face-to-face motivational interviewing		
Focusing: Planning for direction	• Do you perceive any changes in your psychological state compared to before the onset of the disease? • What are the primary sources of psychological distress that you are currently experiencing? • How do you believe your current mental state may impact the progression of the disease? • In what ways do you think making efforts to change negative thoughts, such as feeling a lack of control over diet, excessive worry affecting sleep, and persistent hopelessness about the future. • Which aspects do you believe have been influenced in your life or work following these changes? • If given an opportunity to choose one aspect for improvement, which area would you select? • Who or what holds the utmost importance for you personally?	Three elements: Establishing a specific direction, discovering what matters to the person, and recognizing that this is a gradual and incremental process Core techniques: Open-ended questions, and reflective listening Principles: Expressing empathy, developing conflict, and facilitating self-efficacy Intervention dose: 20 to 30 minutes Location: Inpatient: Quiet, private room in Nephrology Department; Discharged: Prescheduled video interview in a quiet home setting

Week 3: The third face-to-face motivational interviewing		
Calling out: Preparing for change	• Have you ever taken proactive measures to alter your mindset? • How do you perceive yourself in terms of advantages compared to others, including your abilities and resources? • If you were to resolve the issue at hand, how do you anticipate it would impact your mental state? How would it shape your future life? Conversely, if the problem remains unresolved, what significant consequences do you foresee? • Could you kindly share some instances where you have achieved notable success and elaborate on the experiences associated with them?	Core techniques: Affirmation, abstract Principles: Avoid resistance, enhance self-efficacy guidance Intervention dose: 20 to 30 minutes Location: inpatient: Quiet, private room in Nephrology Department; discharged: Prescheduled video interview in a quiet home setting

Week 4: The fourth face-to-face motivational interviewing		
Planning: Bridging to change	• You appear prepared to initiate a transformation, so what are your subsequent objectives? What aspects would you like to witness enhanced? Kindly specify the most pivotal one. • What actions do you believe you can undertake? Do you deem the initial approach satisfactory? Alternatively, what measures do you think other adopt? • What will be your primary course of action? When should this plan be implemented? Who can provide support for your proposed change? • (Confirming the participant's commitment to the scheme) When would you like to take action? How do you perceive the implementation plan? How will you navigate any encountered obstacles? • We can also document these plans as a personal reminder to prevent forgetfulness. Subsequently, it is imperative that you diligently execute the plan on a daily basis. During our next follow-up session, please update me on recent progress and share any insights or reflections.	Five steps: Goal setting, approach selection, plan development, commitment reconfirmation and reinforcement, change support Core techniques: Information exchange, summarization, affirmation Principle: self-efficacy guidance Intervention dose: 20 to 30 minutes Location: inpatient: Quiet, private room in Nephrology Department; discharged: Prescheduled video interview in a quiet home setting

Weeks 5 and 6: The first telephone follow-up guided by motivational interviewing		
Consolidating and strengthening	• How have you been feeling lately? Have you experienced any discomfort? How would you compare your current state to before? • During this period, have you successfully implemented your daily plan in time? Could you kindly share it with me? • (If the patients completed on schedule) You have been doing well every day. What motivates you to persist in your daily routine? Your remarkable progress deserves recognition, and I would like to share your experiences with other patients to inspire them. What are your thoughts on this idea? • (If not completed on schedule) Why were there a few days when you couldn't maintain consistency? Did any emergencies arise? Do you feel that you are deviating further from the goal that was set for yourself? What are your current reflections?	The continuity of the guide, focus, call out, and plan cycle of the initial four interviews can be maintained if the participant fails to adhere to their commitment Intervention dose: 10 to 20 minutes Location: A quiet home setting

Weeks 7 and 8: The second telephone follow-up guided by motivational interviewing		
Consolidating and strengthening	Continue to gain insights into the current condition of patients, ascertain patients' motivations through effective communication, consistently provide encouragement and affirmation to patients, and foster a strong rapport with them.	Intervention dose: 10 to 20 minutes Location: A quiet home setting

Weeks 9 and 10: The third telephone follow-up guided by motivational interviewing		
Consolidating and strengthening	Continue to gain insights into the current condition of patients, ascertain patients' motivations through effective communication, consistently provide encouragement and affirmation to patients, and foster a strong rapport with them.	Intervention dose: 10 to 20 minutes Location: A quiet home setting

Weeks 11 and 12: The fourth telephone follow-up guided by motivational interviewing		
Consolidating and strengthening	Continue to gain insights into the current condition of patients, ascertain patients' motivations through effective communication, consistently provide encouragement and affirmation to patients, and foster a strong rapport with them. Schedule the next qualitative interview.	Intervention dose: 10 to 20 minutes Location: A quiet home setting

**Table 2:** Outcomes and their measurement tools.

Outcomes	Measurement tools	Description	Cronbach's alpha	Timepoints
Self-management behavior	Chronic Kidney Disease Self-Management Instrument (CKD-SM)	• Developed by Lin et al. [29] and revised by Liu et al. [30]	*α* = 0.970	T1, T2, T3, T4
		• 4 dimensions: Self-regulation, problem-solving, social support seeking, and medical adherence		
		• 29 items		
		• 4-point Likert scale		
		• Higher scores, stronger self-management abilities		

Perceived empowerment	Patient Perception of Empowerment Scale (PPES)	• Developed by Lewin and Piper [31] and revised by Yeh et al. [32]	*α* = 0.891	T1, T2, T3, T4
		• 4 dimensions: Information, decision-making, individualization, and self-management		
		• 11 items		
		• 5-point Likert scale		
		• Higher scores, greater perceived empowerment		

Ego depletion	Self-Regulating Fatigue Scale (SRF-S)	• Developed by Nes et al. [33] and revised by Wang et al. [34]	*α* = 0.793	T1, T2, T3, T4
		• 3 dimensions: Cognition, emotion, and behavior		
		• 16 items		
		• 5-point Likert scale		
		• Higher scores, greater degree of ego depletion		

**Table 3:** Baseline sociodemographic characteristics, disease information, and outcome variables for both groups (*n* = 70).

Characteristics/variables	Intervention group (*n* = 35)	Control group (*n* = 35)	*t*/*χ*^2^/*Z*	*p* value
Age, mean ± SD	40.91 ± 9.95	44.83 ± 9.66	−1.670	0.100
Gender, *n* (%)			0.233	0.629
Male	19 (54.3)	21 (60.0)		
Female	16 (45.7)	14 (40.0))		
Marital status, *n* (%)			1.296	0.255
Married	25 (71.4)	29 (82.9)		
Single/divorced/widowed	10 (28.6)	6 (17.1)		
Educational level, *n* (%)			2.719	0.257
Junior high school or below	10 (28.6)	9 (25.7)		
High school	15 (42.9)	21 (60.0)		
College or above	10 (28.5)	5 (14.3)		
Working or not, *n* (%)			1.609	0.205
On job	31 (88.6)	27 (77.1)		
Jobless	4 (11.4)	8 (22.9)		
Residence, *n* (%)			0.254	0.615
Cities and towns	24 (68.6)	22 (62.9)		
Villages	11 (31.4)	13 (37.1)		
Living status, *n* (%)			0.402	0.526
Living with family	28 (80.0)	30 (85.7)		
Living alone	7 (20.0)	5 (14.3)		
Payment methods for medical expenses, *n* (%)		0.215	0.643	
Medical insurance	33 (94.3)	32 (91.4)		
Self-paying	2 (5.7)	3 (8.6)		
Per capita monthly household income (RMB^a^), *n* (%)		2.586	0.274	
< 2000	8 (22.9)	14 (40.0)		
2000∼5000	19 (54.2)	16 (45.7)		
> 5000	8 (22.9)	5 (14.3)		
Duration of disease, *n* (%)			0.245	0.621
3∼12 months	12 (34.3)	14 (40.0)		
> 12 months	23 (65.7)	21 (60.0)		
Stage of CKD, *n* (%)			0.567	0.753
Stage 1	19 (54.3)	18 (51.4)		
Stage 2	13 (37.1)	12 (34.3)		
Stage 3	3 (8.6)	5 (14.3)		
Review frequency, *n* (%)			0.057	0.811
1∼3 months	17 (48.6)	18 (51.4)		
> 3 months	18 (51.4)	17 (48.6)		
Number of hospitalizations, *n* (%)			0.432	0.806
1 time	23 (65.7)	22 (62.9)		
2∼5 times	7 (20.0)	6 (17.1)		
> 5 times	5 (14.3)	7 (20.0)		
BMI, *n* (%)			3.113	0.375
< 18.5	3 (8.6)	2 (5.7)		
18.5∼23.9	17 (48.6)	12 (34.3)		
24∼27.9	9 (25.7)	16 (45.7)		
≥ 28	6 (17.1)	5 (14.3)		
Self-management behavior, mean ± SD	84.26 ± 20.09	82.89 ± 20.93	0.280	0.781
Self-regulation	34.06 ± 7.37	34.34 ± 7.74	−0.158	0.875
Problem-solving	25.86 ± 7.04	24.57 ± 7.18	0.756	0.452
Social support seeking	12.69 ± 3.72	11.74 ± 4.38	0.971	0.335
Medical adherence	11.66 ± 3.69	12.23 ± 3.59	−0.656	0.514
Ego depletion, mean ± SD	42.70 ± 7.04	41.74 ± 8.30	0.372	0.711
Cognition	17.67 ± 2.89	17.32 ± 2.87	0.431	0.668
Emotion	13.12 ± 3.04	13.19 ± 3.53	−0.227	0.821
Behavior	11.91 ± 2.61	11.23 ± 2.96	0.847	0.400
Perceived empowerment, mean ± SD	42.57 ± 6.53	40.77 ± 6.60	1.147	0.255
Information	11.14 ± 2.17	10.49 ± 2.36	1.213	0.229
Decision-making	8.26 ± 1.12	8.34 ± 1.47	−0.274	0.785
Individualization	10.63 ± 2.85	10.03 ± 2.80	0.888	0.378
Self-management	12.54 ± 1.27	11.91 ± 1.56	1.850	0.069
Scr (μmol/L), mean ± SD	78.63 ± 28.63	82.54 ± 30.51	−0.554	0.582
BUN (mmol/L), mean ± SD	6.09 ± 1.89	7.20 ± 2.75	−1.966	0.054
BUA (μmol/L), mean ± SD	373.86 ± 91.95	385.26 ± 85.88	−0.536	0.594
eGFR (ml/min/1.73 m^2^), mean ± SD	97.87 ± 22.62	94.87 ± 23.52	0.545	0.587

*Note:* Scr, serum creatinine.

Abbreviations: BMI, body mass index; BUA, blood uric acid; BUN, blood urea nitrogen; eGFR, estimated glomerular filtration rate; SD, standard deviation.

^a^Considering USD1 = RMB 7.3.

**Table 4:** The comparison of outcome variables between intervention and control groups: generalized estimating equation analysis (*n* = 70).

	Within-group differences						Between-group differences				Effect size (Cohen *d*)
	Intervention group (*n* = 35)			Control group (*n* = 35)			At each time point		Changes from baseline (95% CI)		
	Mean (95% CI)	Changes from baseline (95% CI)	*p* value	Mean (95% CI)	Changes from baseline (95% CI)	*p* value	Adjusted mean difference (95% CI)	*p* value	Adjusted mean difference (95% CI)	*p* value	
Self-management behavior											
T1	84.26 (77.70–90.82)	NA	NA	82.89 (76.05–89.72)	NA	NA	1.37 (−13.72–16.47)	1.000	NA	NA	NA
T2	101.14 (98.22–104.07)	16.89 (8.17–25.60)	< 0.001^∗∗^	81.49 (75.43–87.54)	−1.40 (−5.87–3.07)	1.000	19.66 (8.94–30.37)	< 0.001^∗∗^	18.26 (6.41–30.10)	< 0.001^∗∗^	0.90
T3	106.62 (104.36–108.87)	22.36 (13.17–31.55)	< 0.001^∗∗^	77.66 (71.55–83.77)	−5.23 (−12.10–1.64)	0.487	25.13 (14.83–35.20)	< 0.001^∗∗^	23.73 (12.26–35.20)	< 0.001^∗∗^	1.35
T4	106.67 (105.35–107.98)	22.41 (12.61–32.21)	< 0.001^∗∗^	74.26 (68.47–80.11)	−8.63 (−16.01–1.24)	0.007^∗∗^	32.41 (22.85–41.96)	< 0.001^∗∗^	23.78 (12.69–34.87)	< 0.001^∗∗^	1.52

Ego depletion											
T1	42.83 (40.59–45.07)	NA	NA	42.17 (39.59–44.75)	NA	NA	0.66 (−2.76–4.07)	0.706	NA	NA	NA
T2	33.71 (31.70–35.73)	−9.11 (−11.36–6.87)	< 0.001^∗∗^	43.74 (40.94–46.54)	1.57 (−0.43–3.57)	0.228	−10.03 (−13.48–6.58)	< 0.001^∗∗^	−8.46 (−13.67–3.25)	< 0.001^∗∗^	1.46
T3	30.82 (29.28–32.37)	−12.01 (−14.42–9.59)	< 0.001^∗∗^	44.25 (41.15–47.35)	2.08 (−0.41–4.57)	0.166	−13.43 (−16.89–9.96)	< 0.001^∗∗^	−11.35 (−16.13–6.56)	< 0.001^∗∗^	1.92
T4	28.82 (27.62–30.02)	−14.01 (−16.55–11.47)	< 0.001^∗∗^	45.58 (42.50–48.66)	3.41 (0.60–6.22)	0.008^∗∗^	−16.76 (−20.06–13.46)	< 0.001^∗∗^	−13.35 (−17.88–8.82)	< 0.001^∗∗^	2.37

Perceived empowerment											
T1	42.57 (40.44–44.70)	NA	NA	40.77 (38.62–42.93)	NA	NA	1.80 (−3.03–6.63)	1.000	NA	NA	NA
T2	46.54 (44.84–48.25)	3.97 (1.48–6.47)	< 0.001^∗∗^	39.86 (38.00–41.71)	−0.91 (−2.31–0.49)	0.509	6.69 (2.68–10.70)	< 0.001^∗∗^	5.77 (1.39–10.15)	0.001^∗∗^	0.74
T3	50.18 (49.23–51.12)	7.61 (5.21–10.00)	< 0.001^∗∗^	38.69 (37.00–40.37)	−2.08 (−4.16–0.01)	0.049^∗^	11.49 (8.41–14.57)	< 0.001^∗∗^	9.41 (5.65–13.16)	< 0.001^∗∗^	1.47
T4	49.76 (48.77–50.74)	7.19 (4.95–9.43)	< 0.001^∗∗^	38.52 (36.86–40.18)	−2.26 (−4.80–0.29)	0.118	11.24 (8.16–14.32)	< 0.001^∗∗^	8.99 (5.21–12.77)	< 0.001^∗∗^	1.43

Scr (μmol/L)											
T1	78.63 (69.28–87.98)	NA	NA	83.06 (73.03–93.08)	NA	NA	−4.43 (−26.27–17.42)	1.000	NA	NA	NA
T2	79.57 (71.26–87.88)	0.94 (−7.48–5.59)	1.000	85.11 (75.52–94.71)	2.06 (−6.20–10.32)	1.000	−5.54 (−25.78–14.69)	1.000	−3.49 (−24.24–17.27)	1.000	0.04
T3	78.15 (69.46–86.84)	−0.48 (−7.10–6.14)	1.000	89.31 (75.93–102.70)	6.26 (−4.03–16.54)	1.000	−11.17 (−36.60–14.27)	1.000	−4.91 (−26.05–16.23)	1.000	0.23
T4	78.03 (69.42–86.64)	−0.60 (−7.37–6.18)	1.000	87.71 (76.22–99.20)	4.65 (−3.50–12.80)	1.000	−9.68 (−32.57–13.21)	1.000	−5.03 (−26.09–16.04)	1.000	0.18

BUN (mmol/L)											
T1	6.09 (5.47–6.70)	NA	NA	7.21 (6.31–8.10)	NA	NA	−1.11 (−2.85–0.62)	1.000	NA	NA	NA
T2	6.94 (6.13–7.75)	0.85 (−0.42–2.12)	1.000	7.36 (6.45–8.26)	0.15 (−1.42–1.71)	1.000	−0.42 (−2.36–1.53)	1.000	−0.27 (−2.20–1.66)	1.000	0.29
T3	6.70 (6.00–7.39)	0.61 (−0.34–1.55)	1.000	8.00 (6.80–9.20)	0.79 (−1.17–2.75)	1.000	−1.30 (−3.51–0.91)	1.000	−0.51 (−2.31–1.30)	1.000	0.07
T4	6.66 (5.90–7.42)	0.57 (−0.42–1.58)	1.000	7.58 (6.58–8.58)	0.37 (−1.37–2.11)	1.000	−0.92 (−2.92–1.08)	1.000	−0.55 (−2.42–1.32)	1.000	0.08

BUA (μmol/L)											
T1	373.86 (343.83–403.88)	NA	NA	384.49 (358.26–410.71)	NA	NA	−10.63 (−74.16–52.90)	1.000	NA	NA	NA
T2	378.63 (349.75–407.51)	4.77 (−43.23–52.77)	1.000	385.49 (355.34–415.64)	1.00 (−56.61–58.61)	1.000	−6.86 (−73.40–59.68)	1.000	−5.86 (−68.03–56.31)	1.000	0.04
T3	390.71 (366.85–414.56)	16.85 (−40.45–74.15)	1.000	375.00 (347.39–402.61)	−9.49 (−58.35–39.38)	1.000	15.71 (−42.45–73.86)	1.000	6.22 (−50.28–62.72)	1.000	0.30
T4	381.09 (356.95–405.23)	7.23 (−44.28–58.75)	1.000	370.74 (348.55–392.93)	−13.74 (−68.33–40.84)	1.000	10.35 (−41.90–62.60)	1.000	−3.39 (−60.20–53.41)	1.000	0.24

eGFR (ml/min/1.73 m^2^)											
T1	97.87 (90.49–105.26)	NA	NA	94.29 (86.55–102.04)	NA	NA	3.57 (−13.49–20.63)	1.000	NA	NA	NA
T2	96.64 (88.40–104.89)	−1.23 (−10.13–7.67)	1.000	91.41 (83.90–98.92)	−2.89 (−11.82–6.03)	1.000	5.24 (−12.54–23.01)	1.000	2.34 (−15.68–20.37)	1.000	0.07
T3	97.79 (89.57–106.01)	−0.08 (−7.45–7.29)	1.000	85.68 (77.24–94.11)	−8.62 (−16.94–0.30)	0.034^∗^	12.12 (−6.65–30.88)	1.000	3.49 (−14.51–21.50)	1.000	0.37
T4	97.21 (89.29–105.14)	−0.66 (−8.71–7.39)	1.000	86.94 (78.80–95.09)	−7.36 (−15.08–0.37)	0.083	10.27 (−7.84–28.38)	1.000	2.91 (−14.75–20.58)	1.000	0.29

*Note:* Scr, serum creatinine. T1, baseline; T2, after 4 weeks of intervention; T3, immediately postintervention completion; T4, 4 weeks after intervention completion. Adjusted for age, gender, marital status, education level, and monthly household income. The adjusted mean differences between the groups and *p* values were derived from the generalized estimating equations.

Abbreviations: BMI, body mass index; BUN, blood urea nitrogen; BUA, blood uric acid; CI, confidence interval; eGFR, estimated glomerular filtration rate; NA, not applicable; SD, standard deviation.

^∗^ *p* < 0.05.

^∗∗^ *p* < 0.01.

## Data Availability

The data that support the findings of this study are available from the corresponding authors upon reasonable request.

## Supporting Information

Supporting Information 1 Supporting Information 1: Two rounds of the Delphi method for the psychological empowerment–based motivational interviewing program.

Supporting Information 2 Supporting Information 2: TIDieR checklist.

Supporting Information 3 Supporting Information 3: Table S1: Semistructured interview guide for the qualitative phase. Table S2: Sociodemographic characteristics of the participants (*n* = 14). Table S3: Themes, subthemes, and supporting quotes of the semistructured interviews (*n* = 14). Table S4: COREQ checklist.
