# Supplementary material for: Effects of Psychological Empowerment–Based Motivational Interviewing Program on Self-Management Behavior in Patients With Early Chronic Kidney Disease: A Mixed Methods Study
Source: J Nurs Manag. 2025 Nov 21;2025:6822744. doi: 10.1155/jonm/6822744 (PMC12662694; doi:10.1155/jonm/6822744)
Supplement: Supporting Information 1 — Two rounds of the Delphi method for the psychological empowerment–based motivational interviewing program. [file 6822744.f1.docx]

**Supplementary material 1**

**Two rounds of Delphi method for the psychological empowerment-based motivational interviewing program**

**1. Methods**

*1.1. Development of the expert consultation questionnaire*

Based on the preliminary intervention program, the first-round expert consultation questionnaire was developed to solicit experts’ opinions and suggestions. The expert consultation questionnaire consisted of the following four parts: (1) questionnaire instructions: introducing the research purpose and background, content of the consultation form, filling requirements, specified time, and contact information of the researchers; (2) expert basic information survey: including the expert’s age, years of work experience, educational background, professional title, research field, workplace, etc.; (3) main body of the consultation form: the preliminarily developed intervention program, requesting experts to rate the importance of each indicator and to suggest modifications or additions; (4) expert familiarity and judgment basis: requesting experts to self-evaluate their familiarity with the consultation content and the basis of their judgments.

The second-round expert consultation questionnaire was revised, with items deleted or added based on the opinions and suggestions from the first round. Modifications were highlighted using underlining and bold font to facilitate clearer review by the experts.

*1.2. Selection of consulting experts*

The principles for selecting experts in the Delphi method are representativeness, diversity, and authority. Experts with relevant experience from various disciplines related to the research topic were selected, and participation was voluntary to ensure the objectivity and reliability of the consultation results. The Delphi method typically requires 5–20 experts. After discussion within the research team, 12 experts in related fields were selected for consultation using an empirical selection approach.

*1.3. Expert inclusion criteria*

Bachelor’s degree or higher; intermediate professional title or higher; engaged in clinical, nursing, or psychological work related to nephrology, or in psychological work for other chronic diseases for ≥10 years; voluntary participation in this study, with a commitment to complete at least two rounds of expert consultation.

*1.4. Implementation steps of expert consultation*

Two rounds of expert consultation were conducted. Questionnaires were primarily distributed via email and WeChat. Experts were requested to complete the questionnaire within one week after receipt. They rated the importance of each indicator (1=very unimportant, 2=unimportant, 3=moderately important, 4=important, 5=very important), provided modification suggestions in the “Revision Comments” column, added items in the “Add Items” column, and offered other opinions and suggestions in the “Other Suggestions” section. After the first round, the research team revised the questionnaire based on the experts’ feedback, with changes clearly marked, to form the second-round expert consultation questionnaire. The interval between the two rounds was two weeks.

*1.5. Statistical analysis*

The collected data were entered and analyzed by two individuals using Excel 2019 and SPSS 26.0. Descriptive analysis was used for the experts’ general information. The positive coefficient of experts was described using the questionnaire recovery rate for both rounds, the proportion of experts completing the consultation within the required time, and the proportion of experts raising questions. A questionnaire recovery rate > 50% indicates good expert enthusiasm, and > 70% indicates very high enthusiasm. The arithmetic mean and full score rate of the importance ratings were used to describe the concentration of expert opinions on the relative importance of each indicator. The mean should be ≥3.5; a higher full score rate indicates that more experts gave a full score to the item, implying greater importance. Kendall’s concordance coefficient (W) and the coefficient of variation (CV) were used to describe the coordination degree of expert opinions. The W value ranges from 0 to 1; a higher score indicates greater consistency among experts on the indicator content. A smaller CV value indicates higher consistency among expert opinions; CV should be <0.25. The expert authority coefficient (Cr) was used to describe the authority level. Cr is generally determined by two factors: the basis of the expert's judgment (Ca) and the coefficient of familiarity with the indicators (Cs). Cr = (Ca + Cs)/2. A higher Cr indicates greater expert authority; Cr > 0.7 is generally considered acceptable, and > 0.8 indicates high reliability. The quantitative assignments for the experts’ judgment basis and degree of influence in this study are shown in **Table S1**. The quantitative assignments for the experts' familiarity with the consultation content are shown in **Table S2**.

**Table S1**

Quantitative assignment of the degree of influence of expert judgment basis.

| Judgment basis | Degree of influence | | |
| --- | --- | --- | --- |
|  | Large | Medium | Small |
| Theoretical analysis | 0.3 | 0.2 | 0.1 |
| Practical experience | 0.5 | 0.4 | 0.3 |
| Literature | 0.1 | 0.1 | 0.1 |
| Intuitive feeling | 0.1 | 0.1 | 0.1 |

**Table S2**

Quantitative assignment of expert familiarity

| Familiarity level | Very familiar | Relatively familiar | Moderately familiar | Somewhat unfamiliar | Unfamiliar |
| --- | --- | --- | --- | --- | --- |
| Assignment | 1.0 | 0.8 | 0.6 | 0.4 | 0.2 |

**2. Results**

*2.1. Characteristics of the experts*

A total of 12 experts participated in the Delphi expert consultation. The general information of the experts is shown in **Table S3**.

**Table S3**

Characteristics of the experts.

| Expert No. | Gender | Age(years) | Education | Professional title | Department | Years in Profession | Professional Field |
| --- | --- | --- | --- | --- | --- | --- | --- |
| 1 | Male | 45 | PhD | Associate senior | Neurology | 15 | Neurology |
| 2 | Female | 50 | PhD | Senior | Psychology | 32 | Clinical psychology |
| 3 | Male | 43 | PhD | Associate senior | Psychology | 16 | Psychometrics |
| 4 | Female | 42 | PhD | Associate senior | Nephrology | 18 | Pathogenesis of renal diseases |
| 5 | Female | 39 | Bachelor | Intermediate | Nephrology | 12 | Renal nursing |
| 6 | Female | 43 | Bachelor | Associate senior | Nephrology | 25 | Nursing management for CKD |
| 7 | Male | 40 | Master | Associate senior | Nephrology | 13 | Prevention and treatment of CKD |
| 8 | Female | 40 | Bachelor | Intermediate | Nephrology | 17 | Renal nursing |
| 9 | Female | 36 | Bachelor | Intermediate | Nephrology | 13 | Renal nursing |
| 10 | Male | 48 | PhD | Senior | Psychology | 26 | Applied psychology |
| 11 | Female | 39 | PhD | Associate senior | \| Psychiatry \| \| --- \| | 14 | Pathogenesis and treatment of common mental disorders |
| 12 | Female | 54 | PhD | Senior | Psychology | 31 | Military psychology |

*2.2. Positive coefficient of experts*

Two rounds of Delphi expert consultation were conducted. The first-round questionnaire recovery rate was 70.59%, and the second round was 100%. The proportion of experts completing the consultation within one week was 66.67% and 83.33%, respectively. The percentage of experts raising questions was 75% and 33.33% in the two rounds, respectively, indicating a high level of enthusiasm among experts for this study.

*2.3. Expert authority degree*

The expert authority degree was calculated based on self-rated judgment basis and familiarity with the content. Ca = 0.83, Cs = 0.87, Cr = (Ca + Cs)/2 = 0.85 > 0.8, indicating high expert authority and reliable results.

*2.4. Concentration of expert opinions*

In the first round of expert consultation, the mean importance scores ranged from 3.92 to 4.75 (all >3.5). The standard deviation for each indicator ranged from 0.00 to 0.90. The full score rate ranged from 8.33% to 91.67%, indicating a high concentration of expert opinions.

In the second round of expert consultation, the mean importance scores ranged from 3.92 to 5.00 (all >3.5). The standard deviation for each indicator ranged from 0.00 to 0.67. The full score rate ranged from 16.67% to 100%, indicating a high concentration of expert opinions.

*2.5. Coordination of expert opinions*

In the first round of expert consultation, the coefficient of variation (CV) for each indicator ranged from 0.06 to 0.20 (all <0.25). The Kendall’s concordance coefficient (*W*) for indicators at all levels was 0.229 (*P*<0.001), and the difference was statistically significant.

In the second round of expert consultation, the CV for each indicator ranged from 0.00 to 0.15 (all <0.25). The *W* for indicators at all levels was 0.387 (*P*<0.001), and the difference was statistically significant.

*2.6. Revisions to the preliminary draft*

Based on the opinions and suggestions raised by the experts and after discussion within the research team, the specific revisions were as follows:

(1) In the secondary indicator “1-1 First month: Intervene once a week, 4 sessions in total, 30–45 minutes per session.” was revised to “1-1 First month: Intervene once a week, 4 sessions in total, 20–30 minutes per session.”

(2) In the secondary indicator “7 Evaluation time: Baseline evaluation before intervention; questionnaire survey one month and three months after intervention; qualitative interview one month after the end of intervention.” was revised to “7 Evaluation time: Baseline evaluation before intervention; questionnaire survey at 4 weeks, 12 weeks, and 4 weeks after intervention completion; qualitative interview 4 weeks after intervention completion.”

(3) In the tertiary indicator “5-1-2: What results do you hope to achieve in terms of your physical and psychological state in the next 3 months?” was revised to “What changes do you hope to see in your physical condition in the next 3 months? And “What results do you hope to achieve in terms of your psychological state in the next 3 months?”

(4) In the tertiary indicators (primarily part *5 Intervention Content*) under “5-2-1: The research team consists of 4 members: 1 attending physician responsible for guiding clinical professional knowledge; 1 head nurse responsible for communicating with patients and families and coordinating work; 1 nursing professor with psychological consultant qualification responsible for quality control throughout the intervention study; 1 nursing postgraduate student responsible for implementing the intervention plan.” was revised to “The research team consists of 8 members: 1 nursing professor with psychological consultant qualification responsible for supervising the execution and quality control throughout the intervention study; 1 associate chief physician responsible for guiding clinical professional knowledge; 3 head nurses (one of whom has psychological consultant qualification) responsible for communicating with patients and families and coordinating work; 3 nursing postgraduate students (one of whom has psychological consultant qualification) responsible for implementing the intervention plan, data collection, and analysis.”

(5) In the tertiary indicator “5-3-1: Primary disease is nephrotic syndrome” was revised to “Primary disease is primary glomerular disease, with a course of ≥3 months.”
