# Supplementary material for: Effects of Psychological Empowerment–Based Motivational Interviewing Program on Self-Management Behavior in Patients With Early Chronic Kidney Disease: A Mixed Methods Study
Source: J Nurs Manag. 2025 Nov 21;2025:6822744. doi: 10.1155/jonm/6822744 (PMC12662694; doi:10.1155/jonm/6822744)
Supplement: Supporting Information 2 — TIDieR checklist. [file 6822744.f2.docx]

**Supplementary material 2**

The TIDieR (Template for Intervention Description and Replication) Checklist*:


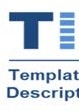

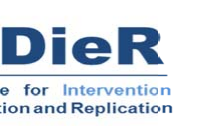


Information to include when describing an intervention and the location of the information

m

| **Item Item** | **Where located **** | |
| --- | --- | --- |
| **number** | Primary paper (page or appendix number) | Other ^†^ (details) |
| **BRIEF NAME**  **1.** Provide the name or a phrase that describes the intervention. | P7-8 |  |
| **WHY** |  |  |
| **2.** Describe any rationale, theory, or goal of the elements essential to the intervention. | P3-4, P7 |  |
| **WHAT** |  |  |
| **3.** Materials: Describe any physical or informational materials used in the intervention, including those | P7-8, P25-28 |  |
| provided to participants or used in intervention delivery or in training of intervention providers. |  |  |
| Provide information on where the materials can be accessed (e.g. online appendix, URL). |  |  |
| **4.** Procedures: Describe each of the procedures, activities, and/or processes used in the intervention, | P25-28 |  |
| including any enabling or support activities. |  |  |
| **WHO PROVIDED** |  |  |
| **5.** For each category of intervention provider (e.g. psychologist, nursing assistant), describe their | P7-8 |  |
| expertise, background and any specific training given. |  |  |
| **HOW** |  |  |
| **6.** Describe the modes of delivery (e.g. face-to-face or by some other mechanism, such as internet or | P7-8, P25-28 |  |
| telephone) of the intervention and whether it was provided individually or in a group. |  |  |
| **WHERE** |  |  |
| **7.** Describe the type(s) of location(s) where the intervention occurred, including any necessary | P25-28 |  |
| infrastructure or relevant features. |  |  |

| **WHEN and HOW MUCH**   1. Describe the number of times the intervention was delivered and over what period of time including P7-8, P25-28 the number of sessions, their schedule, and their duration, intensity or dose.   **TAILORING**   1. If the intervention was planned to be personalised, titrated or adapted, then describe what, why, N/A when, and how.   **MODIFICATIONS**   1. **^ǂ^** If the intervention was modified during the course of the study, describe the changes (what, why, N/A when, and how).   **HOW WELL**   1. Planned: If intervention adherence or fidelity was assessed, describe how and by whom, and if any P8 strategies were used to maintain or improve fidelity, describe them. 2. **^ǂ^** Actual: If intervention adherence or fidelity was assessed, describe the extent to which the N/A   intervention was delivered as planned. |  |
| --- | --- |
| ** **Authors** - use N/A if an item is not applicable for the intervention being described. **Reviewers** – use ‘?’ if information about the element is not reported/not sufficiently reported.  † If the information is not provided in the primary paper, give details of where this information is available. This may include locations such as a published protocol or other published papers (provide citation details) or a website (provide the URL).  ǂ If completing the TIDieR checklist for a protocol, these items are not relevant to the protocol and cannot be described until the study is complete.   - We strongly recommend using this checklist in conjunction with the TIDieR guide (see *BMJ* 2014;348:g1687) which contains an explanation and elaboration for each item. - The focus of TIDieR is on reporting details of the intervention elements (and where relevant, comparison elements) of a study. Other elements and methodological features of studies are covered by other reporting statements and checklists and have not been duplicated as part of the TIDieR checklist. When a **randomised trial** is being reported, the TIDieR checklist should be used in conjunction with the CONSORT statement (see www.consort‐statement.org) as an extension of **Item 5 of the CONSORT 2010 Statement.**   When a **clinical trial protocol** is being reported, the TIDieR checklist should be used in conjunction with the SPIRIT statement as an extension of **Item 11 of the SPIRIT 2013 Statement** (see www.spirit‐statement.org). For alternate study designs, TIDieR can be used in conjunction with the appropriate checklist for that study design (see www.equator‐network.org). |  |
