# Supplementary material for: Effects of Psychological Empowerment–Based Motivational Interviewing Program on Self-Management Behavior in Patients With Early Chronic Kidney Disease: A Mixed Methods Study
Source: J Nurs Manag. 2025 Nov 21;2025:6822744. doi: 10.1155/jonm/6822744 (PMC12662694; doi:10.1155/jonm/6822744)

**Supplementary material 3**

**Table S1**

*Semi-structured interview guide for the qualitative phase.*

| 1.Please describe how do you feel about the program of psychological empowerment-based motivational interviewing? What factors contribute to this perception? |
| --- |
| 2. Has participating in this program influenced your attitude toward the disease? If so, what factors have contributed to this change? |
| 3. In what ways do you feel that you have experienced physical and mental changes compared to before participating in this program? What are the underlying reasons for these changes? |
| 4. Have you identified any particular resources or benefits as a result of engaging in this program? If so, please explain the reasons behind these findings. |
| 5. Would you be interested in partaking in similar positive psychological intervention activities again? And will you recommend this program to others? Why? |

**Table S2**

*Socio-demographic characteristics of the participants (n = 14)*

| **ID** | **Gender** | **Age** | **Educational level** | **Marital status** | **Duration of disease (months)** | **Stage of CKD** |
| --- | --- | --- | --- | --- | --- | --- |
| A | Male | 37 | High school | Married | 10 | 3 |
| B | Male | 38 | Junior high school or below | Married | 12 | 1 |
| C | Female | 38 | Junior high school or below | Married | 70 | 1 |
| D | Female | 40 | College or above | Single | 78 | 2 |
| E | Male | 33 | Junior high school or below | Single | 10 | 3 |
| F | Female | 50 | College or above | Divorced | 67 | 1 |
| G | Male | 41 | College or above | Married | 20 | 1 |
| H | Male | 30 | College or above | Married | 60 | 2 |
| I | Female | 52 | High school | Married | 9 | 1 |
| J | Male | 32 | High school | Married | 18 | 2 |
| K | Female | 44 | College or above | Married | 44 | 2 |
| L | Female | 28 | College or above | Married | 60 | 1 |
| M | Male | 48 | High school | Married | 10 | 1 |
| N | Female | 63 | High school | Divorced | 12 | 1 |

**Table S3**

*Themes, subthemes and supporting quotes of the semi-structured interviews (n = 14)*

| **Theme** | **Subtheme** | **Supporting quotes** |
| --- | --- | --- |
| **Theme 1:**  **Changes in self-perception** | Enhancement of self-efficacy | Participant A: “I think the communication between you and me in the past few months has really increased my confidence, and I have realized that as long as I do many things, I can obviously change some problems, so why don’t I do it? This process of learning is gradual but invaluable. Now I have learned a lot about the management of chronic diseases, and I still believe I can apply them in my daily life.”  Participant F: “I am still young, I must have the courage to face all this. The road of life is so long, there will always be some difficulties, I think I need to do is to learn to live with the suffering, my current suffering is the problem of chronic kidney disease (chuckling), I have to be a little more confident, calm to face it. To be honest, I feel pretty strong about myself.” |
|  | Reinforcement of self-acceptance | Participant E: “I have now fully accepted the fact that I am ill. After all, it is no use complaining all the time. The most important thing is that I have to make some changes and make some behaviors to cooperate with the doctor to treat this disease. It's better to be cooperative than to say anything. The more positive you are to face it, the more you find that you seem to have inexhaustible energy, which is not a bad thing.”  Participant G: “Chronic kidney disease belongs to the long-term treatment of the disease, I now in addition to the calf a little puffy, the other has no impact on life, now the indicators are normal, or relatively lucky. Whether we are sick or not, we must face the reality and actively cooperate with the treatment. Kidney disease is not a terminal disease. You see me now, regular review, take medicine on time, usually pay more attention to some living habits, in fact, it is good to get used to, there is not much trouble, mentality change is very important.” |
|  | Improvement of self-identity | Participant L: “Love others must first love yourself, although I did not make much contribution to society, but for my own family, is to rely on my husband and me. I can’t leave my family, and my family can't leave me. I must not give up. I must have the courage to live with the disease. Besides, I usually look no different from the disease. I also have to take myself to see the wonderful things in life, so that my life is still full of value, leaving no regrets. Now with this resolution, I also keep pushing myself to learn, exercise, and grow, and I always take responsibility for my own health.”  Participant M: “I also began to help others slowly, in this process, also found my own sense of existence (finished with a smile). From saving myself to helping others, I feel that I have become stronger and stronger. Everyone has the value of survival, and I am still full of expectations for the future, because I still want to travel and see the outside world, so I must first treat the disease well and control it.” |
|  | Accurate comprehension of disease | Participant I: “Now I also think of one thing, since this disease is difficult to completely cure, then I will face it head-on, listen to the doctors and nurses, treat it well, and control the disease. My only idea now is to stick to the treatment, take the medicine well at home, and then come to the hospital regularly for reexamination, to minimize the damage of the kidney disease and control the progress of the disease.”  Participant J: “In the past, I always wanted to escape, and I felt that it hindered my life at ordinary times, and I did not know how to treat it. Now I feel that I can’t avoid it anyway, and it will only get worse if I don’t treat it. And it's not a serious illness. It can be controlled. Usually listen to the lectures of experts, summarize the experience of some patients, combined with their actual situation, find a scientific way of life suitable for my own, maintain a peaceful state of mind, this disease is not difficult to cope with.” |
|  | Enhanced recognition of individual capabilities | Participant F: “In addition to being strong enough, a large part of the power comes from friends around me. Now the hospital is becoming more and more perfect, not only treating our physical diseases, but also providing us with popular science and health education. Nurses usually send some materials about chronic kidney disease, from the psychological point of view, is also a great support to us. Honestly, if you hadn't communicated with me all this time, I don't think my mindset would have changed so quickly. I might still be complaining. But now I believe that everything will be better. I should encourage myself more and want to do more positive and happy things.”  Participant N: “I used to be a teacher, so although I am old, I can do many things in life by myself. I'm also open to new things. Now I often find a lot of knowledge about kidney disease through the Internet and mobile phone, and I go to study it and then use it in my life, which is convenient and effective. Slowly, I seem to be more interested and willing to do it. Through the interview, I also feel that everyone cares about us very much, and we need to adjust and face illness by ourselves. A lot of warmth in life has also encouraged me, making me more enthusiastic and passionate to face such difficulties. My grandson also accompanies me every day to exercise, exercise with me to clock, I do not feel tired.” |
|  | Comprehension of the meaning of life | Participant D: “I am genuinely delighted that my parents change their ways to cook me all kinds of delicious food every day, my husband is very supportive of me in all aspects, and also cares about my body very much, my parents-in-law will not look down on me because I am sick, if I don't take care of my body, it is really a bit impossible, even if it is not for myself, I want to live for them too. Besides, I have plans to have a baby, and I need to get my body in order to try not to interfere with my birth later.”  Participant I: “The doctor's advice and the comfort of my family helped me a lot, and also made me gradually change my mentality. I want to manage all the causes of aggravation. My child is more concerned than me. He will carefully read the popular science materials issued by the hospital, and they will tell me all kinds of knowledge about kidney disease (take out the booklet for me to read). Under the supervision of my family, I strengthen my management every day, and it does not feel like a burden. I feel very motivated, and it is also for our bright future.”  Participant K: “In fact, I think illness also has the meaning of illness, through my disease, I also rethink my life, the focus of life should be shifted from work to life, work is no longer competitive, the so-called honor has not deliberately to strive for. Taking good care of my body is the most important thing. In the past, my focus was only on work, family, or children. I wanted my child to be successful in the college entrance examination, so I gave her all kinds of guarantees, and I had little rest. Now I find that I should pay more attention to my health and take good care of myself. Also, give myself more time to appreciate the little things in life, and take everything else slowly.” |
| **Theme 2:**  **Improvement of emotions** | Reduction in negative emotions | Participant C: “I used to be very anxious, always thinking about if the future will drag my children, my husband will abandon me, our family’s economic situation will work, consider a lot of problems (sighed). Through this interview, I found that I thought the disease was too serious. As long as I had regular review, took medicine on time, and paid more attention to my living habits, it seemed that I was not different from others. I feel that while I am still in the early stage, I can control it, and a positive attitude is still important.”  Participant G: “Now I had almost forgotten that I was a patient, and I was no longer afraid of my illness. In fact, this process is not easy, but after changing my mentality found that it is not so difficult.”  Participant H: “During this time of interview, I felt full of positive energy, and also drove away my bad mood. I think I should not let myself have been decadent, I just got married, I can’t let my wife and children face such a big burden, I can still insist, can also go to work, to live the rest of the life.” |
|  | Elicitation of positive emotions | Participant D: “At least I will not give myself too much pressure now. I am happy every day and feel that all the problems are not problems.”  Participant E: “Now my friends say that I feel a lot more cheerful than before, it is, happy every day is the most important.”  Participant F: “During this time, I feel full of expectation and progress every day. My daughter says I talk a lot more, smile a lot more, and feel more positive (with a noticeably higher pitch). I also know that since I can't get rid of kidney disease, I have to go hand in hand with it in a healthy way. As long as I have the will to fight, I will be able to overcome it.”  Participant J: “I think the main mentality of my own is quite good, more easy to think, but also very optimistic, not to get stuck in rut, I now full of confidence in the future.”  Participant L: “After this period of chat, I also feel that both in the physical and psychological pressure, are much lighter. Now I am more awake, relaxed and light.” |
|  | Courage to articulate emotions | Participant A: “In my usual work or life, I am quite calm. I dare to let go of myself in front of colleagues and family members, and I don't feel constrained. Sometimes when I encounter some problems, I will talk to my friends in time. In fact, I have talked to you a lot about this communication with you, and sometimes I have to let you listen to me complain. I am really sorry (with a laugh), but if I say it out, I will feel much better.”  Participant M: “Sometimes I am in a bad mood, I will go to the park to take a walk, relax the mood, release the pressure, I feel this method is quite suitable for me, sometimes I can regulate my own mood, and do not have to be afraid of affecting the family and friends around me. ” |
| **Theme 3:**  **Establishment of health behavior** | Active learning disease-related knowledge | Participant E: “I am now more and more eager to understand this disease, to understand what I should do right.”    Participant F: “I began to try to learn disease-related knowledge and participate in activities for kidney patients. Gradually, I was willing to share my knowledge with others, hoping to help more kidney patients.”  Participant H: “Under your guidance, I have learned a lot about chronic kidney disease. I also began to learn about kidney disease diet. I took the initiative to follow some public accounts, buy books, and read popular science by some experts, to get along better with kidney disease and reduce the burden on my family.”  Participant J: “Now I can also take the initiative to pay attention to the knowledge of kidney disease, listen to the explanations of experts, communicate with others, and see how others manage the disease.”  Participant N: “Now, I often find a lot of knowledge about chronic kidney disease through the Internet and my mobile phone. I learn it, and then use it in my life. I find that it is not difficult, and it is quite useful.” |
|  | Modification of lifestyle | Participant A: “I can’t eat and drink as casually as before, and I should not pay no attention to rest at ordinary times and always stay up late.”  Participant B: “I now go to work normally every day, and dare not stay up late at night. My work and rest are much more regular than before. In terms of diet, sometimes I can't bear to eat more, but I have consciously controlled it. I used to smoke, and now I know I can’t.”  Participant D: “Now I try to reduce eating out and learn to cook by myself, which is obviously much lighter and very healthy. I used to not exercise, and I would lie down when I came home, and I didn’t want to do anything. Now I basically go out for a walk with my husband or my mother after dinner every day. Sometimes I go out to practice yoga or something on weekends.”  Participant G: “In the first year of illness, due to lack of experience, excessive diet control, my weight loss was rapid. Now my diet is adjusted and more balanced. In addition, I can also insist on measuring blood pressure every day, 5 days a week to exercise, mainly walking, jogging, adhere to early bed and early rise.”  Participant L: “Now I also adapt to regular review, every day to take medicine, pay attention to diet, adhere to exercise life, in fact, used to is also very healthy.”  Participant M: “I also slowly began to adjust the state of mind, regular work and rest, get up early every day to run, adhere to three meals a day on time to eat, I will also according to some diet of kidney disease collocation.” |
|  | Development of interests and hobbies | Participant C: “I used to work very busy, now I can do something I like. I have not found a job at present, usually at home like knitting wool, I can knit anything, clothes, hats, shoes, flowers, all kinds of small animals, as long as you can think of, I can knit. I now sell these in the circle of friends, business is very good. I never expected that my own hobby would actually turn out to be a way to earn money. I find that sometimes don't set ourself too rigid. I can do all this from home now, and I can also make sure to get enough rest and take care of my two kids, and everything is going pretty smoothly.”  Participant K: “In the future, I will also take more time to appreciate the small joys of life and handle other things at a leisurely pace. Every weekend, I go to the flower and bird market to buy some flowers or potted plants, and then arrange them myself at home. I also plan to take a class to learn how to arrange flowers. I really enjoy flowers and plants.” |

**Table S4**

*COREQ (consolidated criteria for reporting qualitative research) checklist*


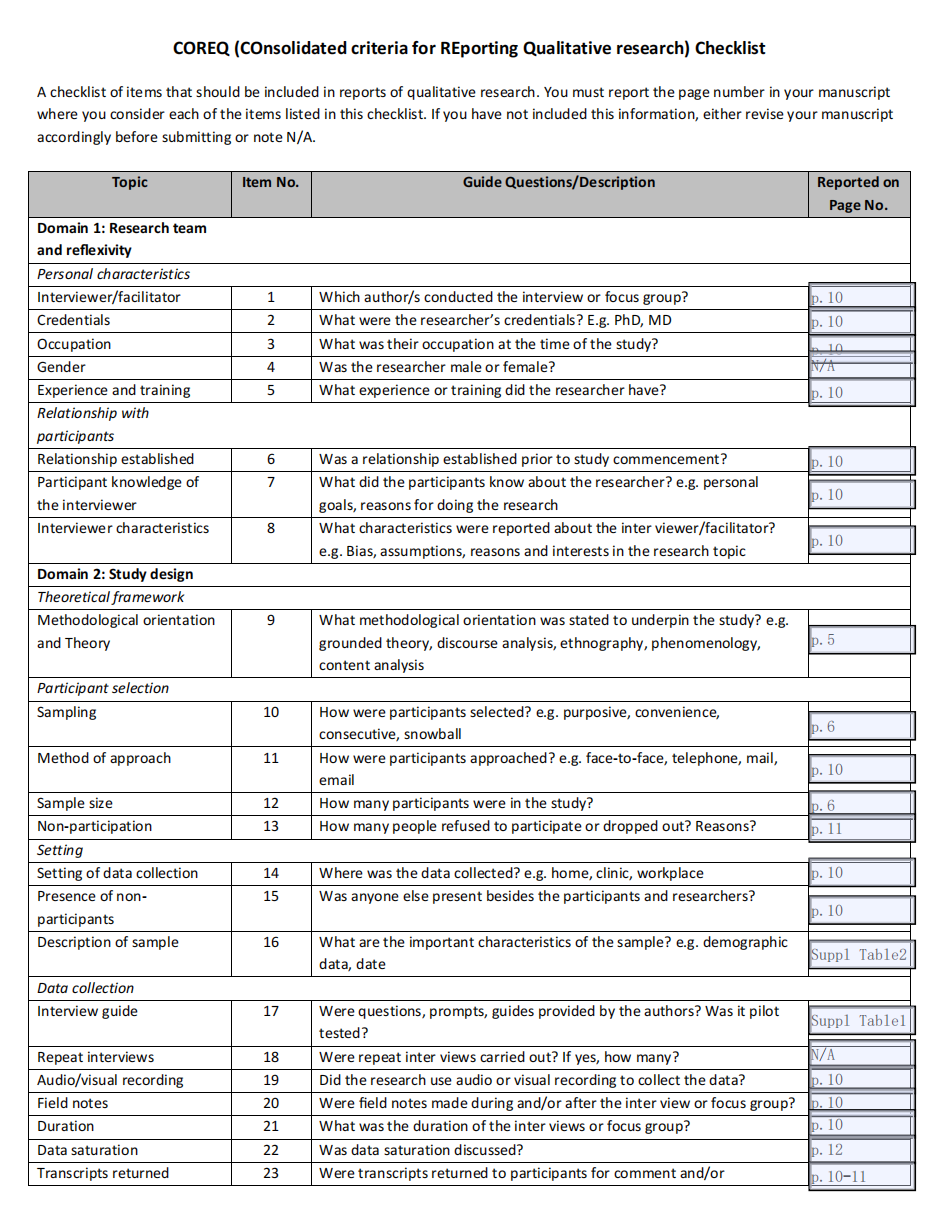


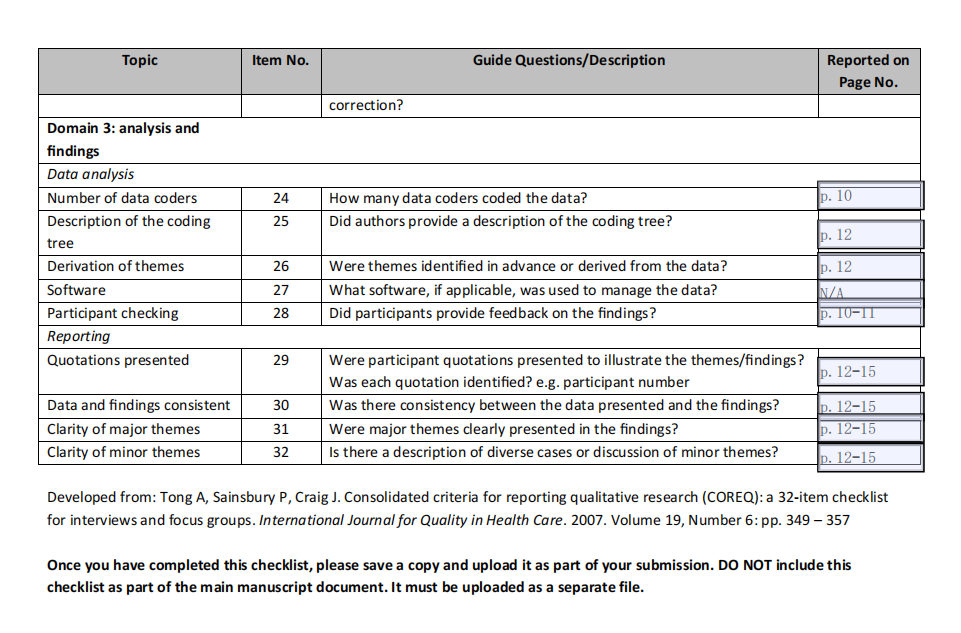

Supplement: Supporting Information 3 — Table S1: Semistructured interview guide for the qualitative phase. Table S2: Sociodemographic characteristics of the participants (n = 14). Table S3: Themes, subthemes, and supporting quotes of the semistructured interviews (n = 14). Table S4: COREQ checklist. [file 6822744.f3.docx]
